# Severe bullous erythema multiforme with atypical mucosal involvement: a rare clinical image

**DOI:** 10.11604/pamj.2025.52.118.47430

**Published:** 2025-11-20

**Authors:** Pooja Kasturkar, Kavita Gomase

**Affiliations:** 1 Department of Mental Health Nursing, Smt Radhikabai Meghe Memorial College of Nursing, Datta Meghe Institute of Higher Education and Research, Sawangi (M) Wardha, India,; 2 Department of Obstetrics and Gynaecological Nursing, Smt Radhikabai Meghe Memorial College of Nursing, Datta Meghe Institute of Higher Education and Research, Sawangi (M) Wardha, India

**Keywords:** Severe bullous erythema multiforme, hypersensitivity reaction, amoxicillin-clavulanic acid allergy, erythematous macules, mucosal necrosis, genital mucosal

## Image in medicine

A three-year-old girl who weighed 8.4kg and was 80cm tall was brought in with weakness, drowsiness, and a fever. Injection of Augmentin (Amoxicillin-clavulanic acid) was prescribed to her. She experienced a significant hypersensitive reaction within 24 hours, resulting in erythematous macules, bullae on the face, trunk, and extremities, and widespread involvement of the oral mucosa, which hindered oral intake. She had no known allergies, no recent pharmaceutical exposure. Laboratory investigations showed elevated erythrocyte sedimentation rate (ESR) and C-reactive protein (CRP), with a negative autoimmune panel. Serological tests confirmed *Mycoplasma pneumoniae* infection via immunoglobulin M (IgM) positivity. Skin biopsy revealed subepidermal bullae with keratinocyte necrosis and perivascular lymphocytic infiltration, confirming bullous erythema multiforme. The case was diagnosed as severe bullous erythema multiforme with atypical mucosal involvement, resembling paediatric autoimmune blistering diseases or severe bacterial/viral stomatitis. Treatment included systemic corticosteroids (methylprednisolone 1 mg/kg/day), hydration, pain management, and supportive care. Azithromycin was started for *Mycoplasma pneumoniae*. As her condition worsened with severe oral ulcerations and extensive blistering, intravenous immunoglobulin (IVIG) (2g/kg) was given on day 5, leading to significant improvement within 72 hours. Supportive care included antiseptic mouthwash, petroleum jelly, oral morphine for pain, parenteral nutrition, and ophthalmologic monitoring. Dapsone (1 mg/kg/day) helped reduce new blister formation. Complete mucosal healing occurred over three weeks, allowing normal eating. At a six-month follow-up, no long-term complications were noted. The reaction was likely due to immune system immaturity, genetic predisposition, and infection-triggered hypersensitivity. This case highlights the importance of early diagnosis, prompt treatment, and multidisciplinary care in managing drug-induced severe cutaneous reactions in children.

**Figure 1:**
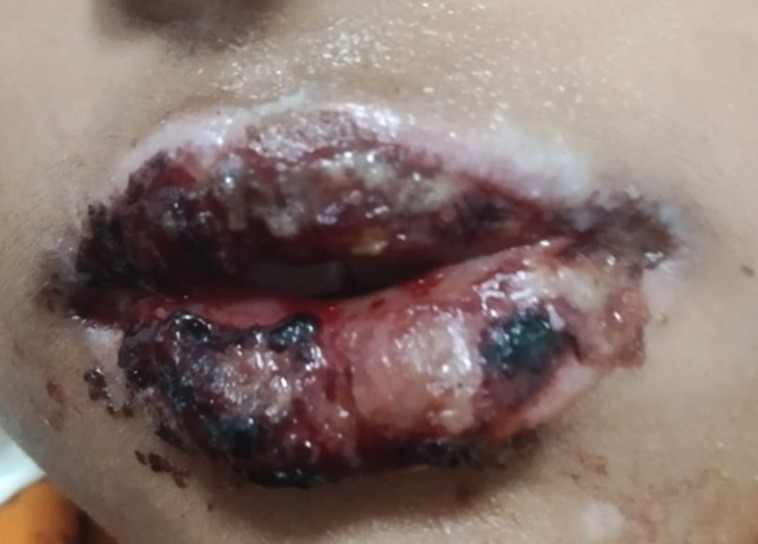
severe bullous erythema multiforme with atypical mucosal involvement

